# Estradiol Enhances CD4^+^ T-Cell Anti-Viral Immunity by Priming Vaginal DCs to Induce T_h_17 Responses via an IL-1-Dependent Pathway

**DOI:** 10.1371/journal.ppat.1005589

**Published:** 2016-05-05

**Authors:** Varun C. Anipindi, Puja Bagri, Kristy Roth, Sara E. Dizzell, Philip V. Nguyen, Christopher R. Shaler, Derek K. Chu, Rodrigo Jiménez-Saiz, Hong Liang, Stephanie Swift, Aisha Nazli, Jessica K. Kafka, Jonathan Bramson, Zhou Xing, Manel Jordana, Yonghong Wan, Denis P. Snider, Martin R. Stampfli, Charu Kaushic

**Affiliations:** 1 Department of Pathology and Molecular Medicine, McMaster University, Hamilton, Ontario, Canada; Thomas Jefferson University, UNITED STATES

## Abstract

Clinical and experimental studies have shown that estradiol (E2) confers protection against HIV and other sexually transmitted infections. Here, we investigated the underlying mechanism. Better protection in E2-treated mice, immunized against genital HSV-2, coincided with earlier recruitment and higher proportions of T_h_1 and T_h_17 effector cells in the vagina post-challenge, compared to placebo-treated controls. Vaginal APCs isolated from E2-treated mice induced 10-fold higher T_h_17 and T_h_1 responses, compared to APCs from progesterone-treated, placebo-treated, and estradiol-receptor knockout mice in APC-T cell co-cultures. CD11c^+^ DCs in the vagina were the predominant APC population responsible for priming these T_h_17 responses, and a potent source of IL-6 and IL-1β, important factors for T_h_17 differentiation. T_h_17 responses were abrogated in APC-T cell co-cultures containing IL-1β KO, but not IL-6 KO vaginal DCs, showing that IL-1β is a critical factor for T_h_17 induction in the genital tract. E2 treatment *in vivo* directly induced high expression of IL-1β in vaginal DCs, and addition of IL-1β restored T_h_17 induction by IL-1β KO APCs in co-cultures. Finally, we examined the role of IL-17 in anti-HSV-2 memory T cell responses. IL-17 KO mice were more susceptible to intravaginal HSV-2 challenge, compared to WT controls, and vaginal DCs from these mice were defective at priming efficient T_h_1 responses *in vitro*, indicating that IL-17 is important for the generation of efficient anti-viral memory responses. We conclude that the genital mucosa has a unique microenvironment whereby E2 enhances CD4^+^ T cell anti-viral immunity by priming vaginal DCs to induce T_h_17 responses through an IL-1-dependent pathway.

## Author Summary

Female sex hormones can affect susceptibility and immune responses to infections. While a number of previous studies, including our own, have shown that progesterone and progesterone-derived hormonal contraceptives increase susceptibility and impair immune responses, estradiol protects against sexually transmitted infections. The reason why estradiol is protective remains unknown. In this study, we investigated the effect of estradiol on dendritic cells, specialized immune cells that determine what type of anti-viral cellular immune responses will be mounted following infection with a sexually transmitted virus, HSV-2. Our studies show that estradiol influences dendritic cells in the vaginal tract of mice to initiate unique anti-viral T cell immunity that results in better protection against genital HSV-2 infection. This type of T cell response is unique to the vaginal tract and not found in any other mucosal lining of the body. This is the first study to show directly that estradiol, a female sex hormone, can determine how well the immune system will combat a sexually transmitted viral infection. The information from this study will be very important in understanding what type of immunity can protect women from sexually transmitted infections and how we can use this information to develop better vaccines.

## Introduction

The female sex hormones estradiol (E2) and progesterone (P4) play a key role in controlling development and function of the reproductive tract, but can also regulate susceptibility and immunity to sexually transmitted infections (STIs) [1–3]. A number of clinical and experimental studies have shown that the menstrual cycle, hormonal contraceptives, and exogenous hormones can determine susceptibility to HIV-1, HSV-2 and *C*. *trachomatis* [2, 4–7]. While P4 and P4-based hormonal contraceptives appear to increase susceptibility and transmission to sexually transmitted viruses, E2 is generally considered protective. Studies in macaque models demonstrated that while medroxyprogesterone acetate (MPA), a P4-based contraceptive, enhanced susceptibility to simian immunodeficiency virus (SIV), E2-treatment protected animals against infection [8, 9]. Studies, including our own, have shown that E2, P4 and hormonal contraceptives influence the anti-viral immune responses and protection outcomes, in a murine model of HSV-2 infection [10–15]. Although the mechanism underlying increased susceptibility to HIV-1 in women using hormonal contraceptives has gained much attention, the protective effect of E2 remains under-investigated.

HSV-2 is the predominant cause of genital herpes, one of the most prevalent sexually transmitted infections in the world. Over 530 million people worldwide are seropositive for HSV-2 [16], and genital herpes is a known co-factor in the acquisition and transmission of HIV-1 [16]. Currently, there is no known vaccine for HSV-2, and anti-viral formulations only reduce the incidence and symptoms of recurrences. Attempts to develop vaccines against HSV-2 have failed since the 1980s [7]. The last large-scale clinical trial of a glycoprotein D based vaccine showed no efficacy, except for partial protection in a sub-group of women seronegative for HSV-1 and HSV-2 [17, 18]. These studies emphasize the need to better understand sex-specific immune responses in the reproductive mucosa, in order to develop effective vaccines against sexually transmitted infections.

A number of studies have examined factors that affect anti-viral immunity in the female reproductive tract [2, 19]. Our own studies have demonstrated that intranasal, subcutaneous or intravaginal immunization with live attenuated thymidine kinase deficient (TK^−^) HSV-2, in the presence of P4, led to protection accompanied by excessive genital inflammation and pathology post-challenge [13, 14]. However, immunization in the presence of E2 led to significantly better protection outcomes: better survival without pathology [13–15]. This protective effect of E2 was verified by others, using an HSV-2 subunit-based glycoprotein gD vaccine candidate [10]. Based on these studies, we hypothesized that the differences in protection quality may be due to the influence of sex hormones on the function of antigen presenting cells (APCs), such as dendritic cells (DCs) in the female genital tract. Vaginal DCs have been examined in a limited number of studies. Four groups of Langerhans cells were characterized in the murine vagina by immunohistochemistry: I-A^+^ F4/80^+^, I-A^+^ F4/80^−^, I-A^−^ CD205^+^ and I-A^+^ CD205^−^ [20]. In a separate study, using flow cytometry, CD11c^+^ MHCII^+^ DCs in the vaginal epithelium were identified as CD11b^+^ F4/80^hi^, CD11b^+^ F4/80^int^, and CD11b^−^ F4/80^−^ subsets [21]. The same group also described a network of CD11c^+^ CD11b^+^ MHCII^+^ DCs in the vaginal lamina propria [22]. The frequency and distribution of these immune cells were shown to alter with the stage of the hormone cycle [23]. CD11c^+^ MHCII^+^ DCs in the vaginal epithelium were distributed abundantly during the metestrus and diestrus phases, but were only found sparsely during the estrus phase. Furthermore, Langerhans cells near the lumen were missing during the estrus phase and only found during the diestrus and matestrus phases [22].

Previous studies have shown that vaginal DCs may be key to the development of CD4^+^ T cell responses against HSV-2 [24], and both E2 and P4 can modulate DC phenotype and functions [25, 26]. It is well documented that alterations in DC functions can shape CD4^+^ T cell-mediated adaptive immune responses [27, 28]. For example, IL-12, IL-15, and TNF-α produced by DCs can bias T_h_0 cells towards T_h_1 effectors, while TSLP, IL-33, and IL-25 can lead to T_h_2 responses. Similarly, TGF-β, IL-10, retinoic acid, and the expression of PDL-1 by DCs can prime T regulatory cells, while IL-6, TGF-β, IL-1 and IL-23 can induce T_h_17 differentiation [28, 29]. Therefore, we examined whether E2 can directly influence vaginal DCs to direct the differentiation of CD4^+^ T cells, and consequently alter the profile of anti-viral T cell responses.

The role of T_h_1 effectors in HSV-2 anti-viral immunity has been well-described [30]. In brief, IFN-γ-producing T_h_1 cells are critical, as demonstrated by studies where the depletion of CD4^+^ T cells and neutralization of IFN-γ, compromised protection against HSV-2 [31]; the administration of exogenous IFN-γ restored protection to CD4^+^ T cell deficient mice [32]. T_h_2 cells and Tregs may lack a direct anti-viral role in the HSV-2 mouse model [33], but the latter have been implicated in facilitating the efficient influx of immune cells such as NK cells, DCs and T cells to the vagina post-primary infection [34]. IL-17, primarily produced by T_h_17 cells, is a normal response of the immune system to *C*. *albicans* and *N*. *gonorrheae* infections in the vagina [35, 36]. However, the role of T_h_17 effector responses in viral infections of the genital mucosa has not been clearly defined.

In the current study, we examined the mechanism underlying the enhanced protection outcomes seen under the influence of E2 in the HSV-2 vaccine model. We observed earlier recruitment, and increased proportions, of T_h_17 and T_h_1 effector cells post-challenge in the vagina of E2-treated immunized mice. E2 treatment directly conditioned vaginal CD11c^+^ APCs to induce T_h_17 responses through an IL-1-dependent, but IL-6-independent, pathway. Furthermore, the ability of vaginal CD11c^+^ APCs to induce predominantly T_h_17 responses was distinct compared to APCs from spleen and other mucosa, suggesting that the hormonal conditioning of APCs is unique to the genital mucosa.

## Results

### E2 treatment enhances protection against WT genital HSV-2 challenge

**Fig 1:**
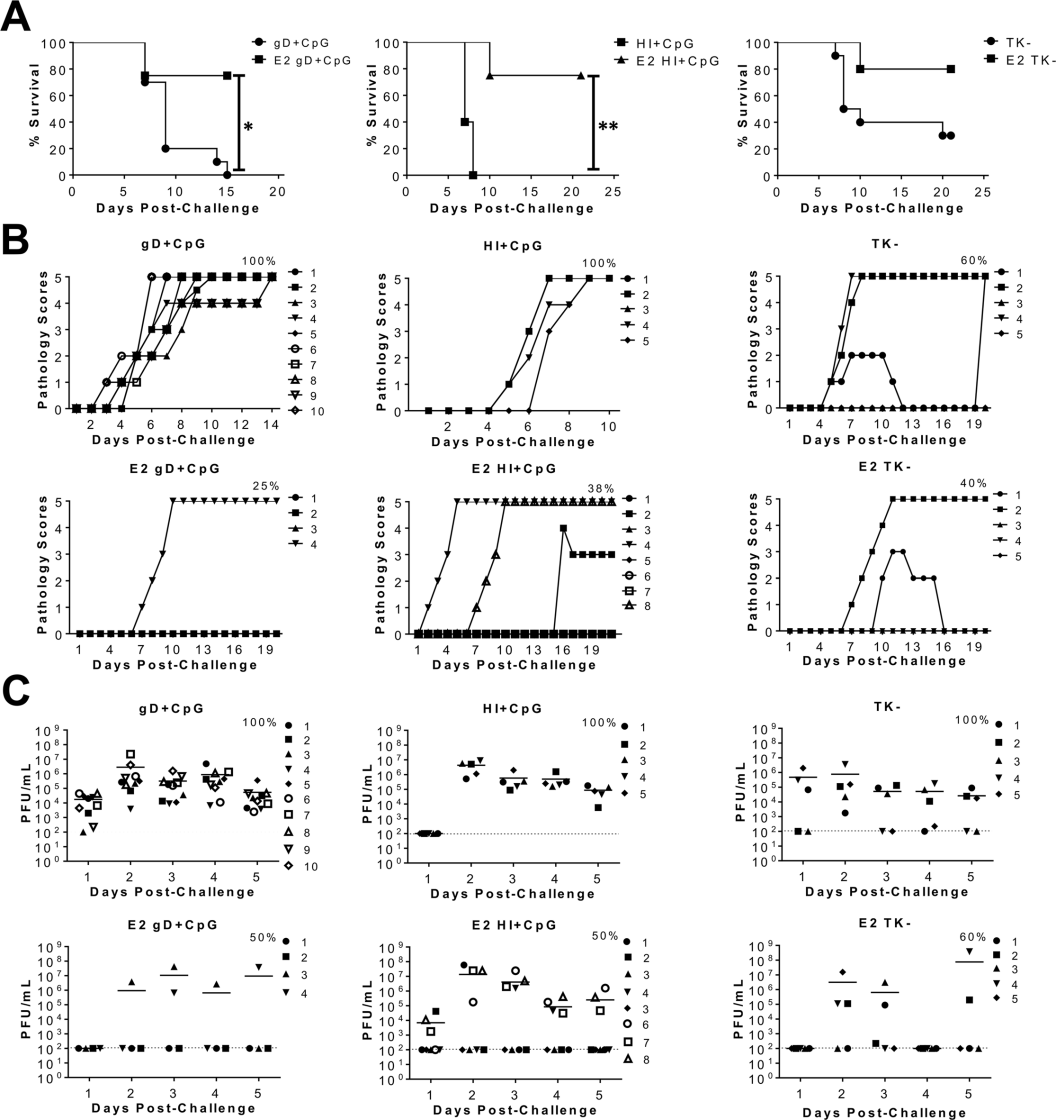
E2 pre-treatment enhances protection against genital HSV-2 challenge in intranasally immunized mice. WT (C57Bl/6) OVX mice treated with E2 or placebo pellets, were intranasally immunized 1 week and 3 weeks later with 1x10^3^ TK^−^ HSV-2, or 5μg HSV-2 gD + 30μg CpG, or 1x10^4^ pfu HI HSV-2 333 + 30μg CpG (n = 5–10 mice/hormone group for each vaccine formulation). Five weeks following the second immunization, all groups of mice were intravaginally challenged with 5x10^3^ pfu/mouse WT HSV-2 333. (A) Survival curves showing the percentage of mice that survived WT HSV-2 challenge in all vaccine formulations. Significance in difference in survival was calculated using the log-rank (Mantel-Cox) test (* p<0.05, ** p<0.01). (B) Pathology scores in these mice were graded on a 1–5 scale as described in the Materials and Methods section, and plotted. Data points superimposed on X-axis indicate mice without genital pathology, and the % indicates maximum number of mice that showed pathology. (C) Vaginal washes were collected for 5 days post-challenge, and HSV-2 viral shedding (bar indicates mean pfu/mL of shed virus) was calculated by conducting viral titrations with a vero-cell based assay. Dashed line indicates the lower detection limit of this assay, and data points on this line indicate undetectable viral shedding. The % indicates maximum number of mice that shed virus between days 1 to 5 post-challenge. Each symbol in B and C represents a single animal and data has been pooled from two separate experiments with similar results.

We have previously shown that intranasal immunization of ovariectomized (OVX) E2-treated mice with live attenuated TK^−^ HSV-2, leads to optimal protection with minimal pathology post-challenge, compared to hormone-naïve OVX controls [14]. We wanted to determine whether enhanced protection would also be seen in E2-treated mice immunized with non-live virus vaccine formulations such as an HSV-2 glycoprotein subunit (gD), or a heat-inactivated (HI) HSV-2. OVX mice implanted with 21-day release E2 pellets, were intranasally immunized with HSV-2 gD + CpG, HI HSV-2 + CpG, or live attenuated TK^−^ HSV-2, and 6 weeks later, challenged intravaginally (IVAG) with a lethal dose of wild type (WT) HSV-2 333. Survival, genital pathology and viral shedding were monitored post-challenge. The control group of OVX mice was implanted with placebo pellets (mock), but underwent similar immunization and challenge. E2 treatment was associated with 80% survival against lethal HSV-2 challenge in the TK^−^ HSV-2 vaccine group, and 75% survival in the gD + CpG and HI HSV-2 + CpG vaccine groups (Fig 1A). However, only 30% of mock controls survived lethal challenge in the TK^−^ HSV-2 vaccine group, while none survived in the gD + CpG or HI HSV-2 + CpG vaccine groups (Fig 1A). Better survival in E2-treated mice corresponded with lower cumulative pathology scores (Table 1), and fewer mice shedding virus on any given day post-challenge (Fig 1C), compared to mock controls (Fig 1B and 1C). Overall, consistent with our previous report [14], these results show that immunization under the influence of E2 enhanced protection by improving survival, and diminishing disease pathology and viral shedding post-challenge, regardless of the vaccine formulation.

**Table 1:** Cumulative pathology scores for HSV-2 pre-exposed WT and IL-17 KO mice challenged with WT HSV-2.

Treatment Group (total # of mice)	PathologyScore	# of mice	# of days	CumulativePathology	Avg. Pathology per Mouse
**gD+CpG (n = 10)**	5	2	9	90	28
	5	1	8	40	
	5	1	7	35	
	5	1	6	30	
	5	3	5	75	
	5	2	1	10	
**E2 gD+CpG (n = 4)**	0	3	14	0	6.25
	5	1	5	25	
**HI+CpG (n = 5)**	5	3	4	60	16
	5	2	2	20	
**E2 HI+CpG (n = 8)**	0	6	10	0	4.38
	5	1	1	5	
	5	1	6	30	
**TK- (n = 5)**	0	2	20	0	28
	5	1	14	70	
	5	1	13	65	
	5	1	1	5	
**E2 TK- (n = 5)**	0	3	20	0	11.2
	3	1	2	6	
	5	1	10	50	

Cumulative pathology is calculated by denoting the number of mice with their maximum pathology score and the number of days that score was observed for each group. This takes into consideration that each mouse in a group can reach varying degrees of pathology through the experiment. Average pathology score per mouse was calculated by dividing the sum of cumulative pathology by total number of mice.

### E2 treatment leads to earlier recruitment and higher proportion of T_h_1 and T_h_17 cells in the vagina

**Fig 2:**
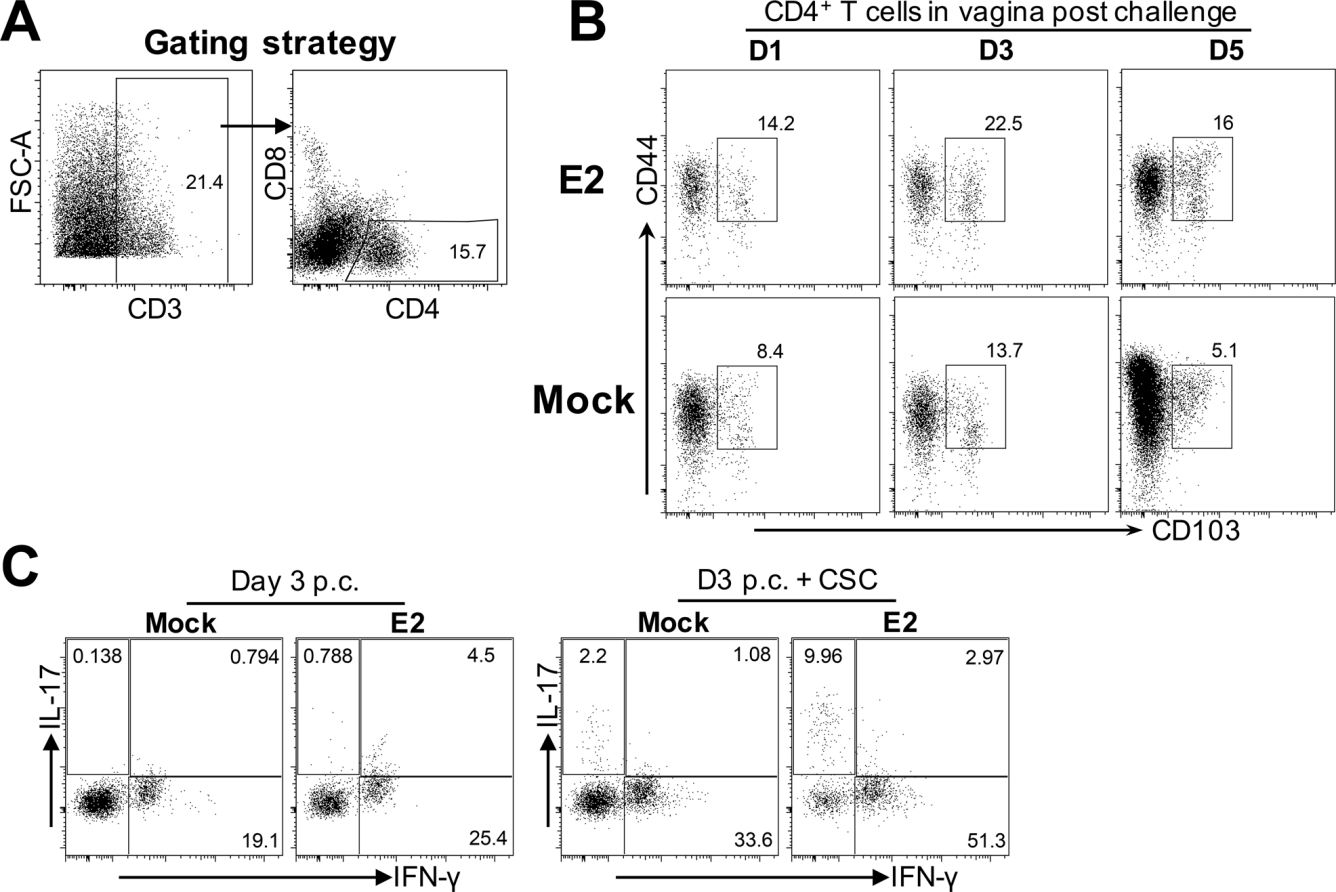
E2 pre-treatment enhances the recruitment of CD103^+^ CD44^+^ CD4^+^ T cells in the vagina, and is related to increased proportions of T_h_1 and T_h_17 cells, post-challenge. WT OVX mice implanted with E2 or placebo pellets (mock) (n = 5–10 mice/group in all three time points: D1, D3 and D5 p.c.), were immunized intranasally with 1x10^3^ pfu/mouse TK^−^ HSV-2, and five weeks later, challenged intravaginally with 5x10^3^ pfu WT HSV-2 333. Vaginal tissues isolated at D1, D3 and D5 post challenge (p.c.), from each group were pooled, processed and stained with a panel of antibodies against CD3, CD4, CD8, CD44, CD103, IL-17 and IFN-γ according to protocols detailed in the Materials and Methods section, and examined by flow cytometry. (A) CD8^-^ CD4^+^ T cells were gated among total CD3^+^ T cells in the vagina. (B) The proportion of mucosal memory CD103^+^ CD44^+^ T cells from tissues isolated on days 1, 3 and 5 p.c. were compared between E2- and placebo (mock)-treated mice. (C) For intracellular staining of IL-17 and IFN-γ, vaginal cells pooled from n = 5 mice per group, at days 1, 3 and 5 p.c, were incubated in the presence of golgi inhibitors alone to examine the *in vivo* response to HSV-2 challenge, or stimulated *in vitro* with cell stimulation cocktail (CSC) containing golgi inhibitors and PMA + ionomycin, for 18h. Intracellular staining for IL-17 and IFN-γ was used to examine the differentiation of CD4^+^ T cells into T_h_17 and T_h_1 cells, respectively. A representative of this data from day 3 p.c. is shown. Data is representative of two independent experiments.

HSV-2–specific IFN-γ-producing T_h_1 CD4^+^ T cells are known to play a critical role in the resolution of intravaginal HSV-2 infection in the mouse model [31, 32]. We wanted to examine whether improved protection in E2-treated mice was related to enhanced T_h_1 responses in the vagina, post-challenge. OVX mice treated with E2 or placebo (mock) pellets were immunized intranasally with TK^−^ HSV-2, and challenged 6-weeks later, intravaginally, with WT HSV-2 333. Vaginal tissue from each group of mice was pooled, and the phenotype of CD4^+^ T cells was examined on days 1, 3 and 5 post-challenge (p.c.). CD4^+^ T cells were gated based on total CD3^+^ cells in the vagina (Fig 2A), and the profile of mucosal memory CD4^+^ T cells (CD44^+^ CD103^+^) was compared between E2 and mock treatment groups. Vaginal tissue from E2-treated mice contained higher proportions of mucosal memory CD4^+^ T cells at all three time points compared to mock controls (Fig 2B). To compare functional differences in the CD4^+^ T cells between these groups, IFN-γ and IL-17 expression in these cells was examined by intracellular staining (ICS). E2-treated mice showed a higher proportion of IFN-γ^+^ T_h_1 and IL-17^+^ T_h_17 cells at earlier time points (days 1 and 3 p.c.) (Fig 2C and Table 2). On day 5 p.c., while a higher proportion of T_h_17 cells was still present in the vagina of E2-treated mice compared to the mock controls, fewer T_h_1 cells were observed in E2-treated mice, likely due to earlier clearance of virus. Following *in vitro* stimulation with PMA and ionomycin, a higher proportion of total T_h_1 and T_h_17 cells was seen in E2-treated mice compared to mock controls at all three time points (D1, D3 and D5 p.c.) (Table 2). Overall, these observations suggest that E2 treatment augments anti-viral responses in the female genital tract by accelerating, and enhancing, T_h_1 and T_h_17 responses post-challenge.

**Table 2:** Cytokine production by CD4^+^ T cells in the vagina post intravaginal HSV-2 challenge.

		D1 p.c.		D3 p.c.		D5 p.c.	
Treatments	Cytokine	Mock	E2	Mock	E2	Mock	E2
***In vivo* challenge alone**	**IL-17** ^**+**^	0.9	2.1	0.9	5.3	0.2	2.8
	**IFN-γ** ^**+**^	7.7	17.8	19.9	29.9	14.1	9.8
	**IL-17** ^**+**^ **IFN-γ** ^**+**^	0.5	1.8	0.8	4.5	0.06	1.7
***In vivo* challenge + *In vitro* stimulation**	**IL-17** ^**+**^	6.5	16.7	3.3	12.9	2.7	20.9
	**IFN-γ** ^**+**^	25.0	39.8	34.7	54.3	30.7	51.7
	**IL-17** ^**+**^ **IFN-γ** ^**+**^	1.2	4.4	1.1	2.9	1.0	6.9

**Mock:** Placebo-treated mice, **E2:** E2-treated mice (n = 5–10 mice/group, pooled tissue), **p.c.:** post-challenge; ***In vivo* challenge:** Cytokine-producing cells (% of total vaginal CD3+ CD4+ cells) at various time points post WT HSV-2 challenge, blocked with golgi inhibitors for 16h without any additional stimulation; ***In vivo* challenge + *in vitro* stimulation:** Cytokine producing cells (% of total vaginal CD3+ CD4+ cells) at various time points post-challenge after *in vitro* stimulation with cell stimulation cocktail containing golgi inhibitors + PMA + ionomycin for 16h. Data representative of two separate experiments with similar results.

### Vaginal cells from E2-treated mice induce IL-17 and IFN-γ from T cells in APC-T cell co-cultures

**Fig 3:**
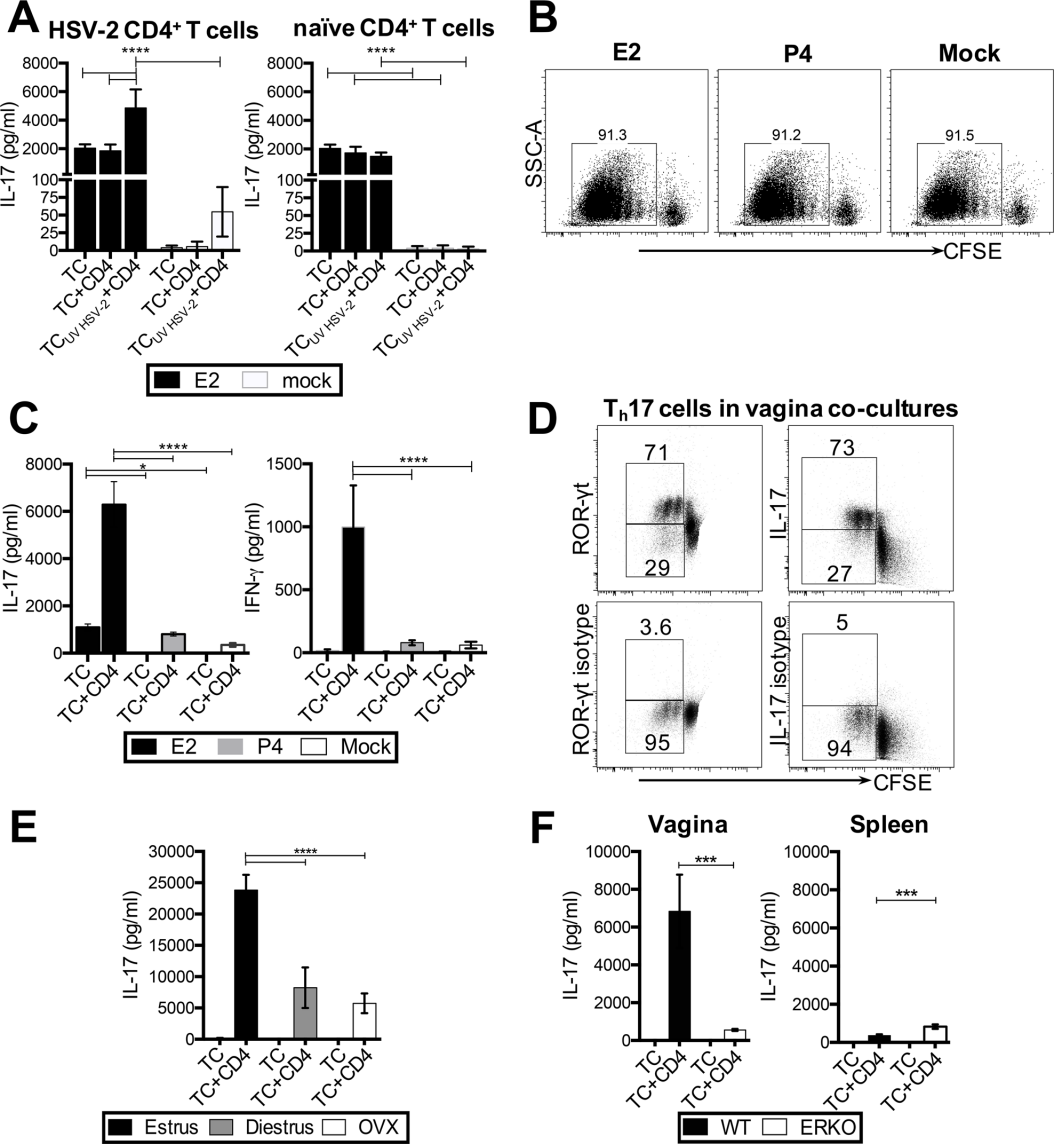
E2 can influence the differentiation of CD4^+^ T cells in vaginal APC-T cell co-cultures. (A) OVX WT mice were implanted with E2 or placebo (mock) pellets, and two weeks later, vaginal tissue cells (TC) were isolated and pulsed with 5x10^5^ pfu/ml UV-inactivated HSV-2 for 16h. These TC were co-cultured for 3.5 days with HSV-2 specific CD4^+^ T cells (TC + HSV-2 CD4) isolated by MACS from the draining lymph nodes and vaginal tracts of HSV-2 immunized and challenged mice. For control co-cultures, naïve CD4^+^ T cells (TC + control CD4) were isolated from the spleen of uninfected OVX mice. IL-17 levels in co-culture supernatants were measured by ELISA. (B) Vaginal tissue cells (TC) (5x10^5^ cells/ml) from OVX mice (n = 6 mice/group) implanted with E2, P4, or placebo pellets (mock) were pulsed with OVA peptide and co-cultured with OT-II Tg CD4^+^ T cells (TC+CD4) (5x10^5^ cells/ml) for 3.5 days. Proliferation of CD4^+^ T cells was compared among co-cultures conducted with TC from E2-, P4- or placebo (mock)-treated mice. (C) IL-17 and IFN-γ levels in co-culture supernatants were measured by ELISA. Data is mean±SD of three individual co-culture wells from one of three separate experiments with similar trends, and significance was calculated by two-way ANOVA (* p<0.05, **** p<0.0001). (D) Intracellular staining of vaginal co-cultures to identify the cellular source of IL-17. On day 2 of co-culture, 2 ul/mL of CSC was added, and 18h later, co-cultures were stained with antibodies against CD3, CD4, IL-17 and IFN-γ, and analyzed on a flow cytometer. (E) Vaginal tissues from ovary-intact mice were pooled depending on the stage of their reproductive cycle (n = 6 mice/stage) (E2-dominant: Estrus, and P4-dominant: Diestrus, and OVX controls), pulsed with OVA-peptide, and co-cultured with OT-II Tg CD4^+^ T cells for 3.5 days. IL-17 levels in co-culture supernatants were measured by ELISA. Data is mean+SD of three individual co-culture wells, representative from one of three separate experiments with similar results, and significance was calculated by two-way ANOVA (**** p<0.0001). (F) IL-17 levels were compared among WT and ERKO vaginal and spleen tissue co-cultures. Data is representative of two separate experiments with similar results, and significance was calculated by two-way ANOVA (*** p = 0.0005).

Since E2-treated mice demonstrated accelerated, and greater T_h_1 and T_h_17 responses, we next wanted to examine whether E2 influences CD4^+^ T cell responses by conditioning vaginal APCs. OVX mice were implanted with E2 or placebo (mock) pellets, and two weeks later, vaginal tissue cells (TC) containing all local APCs were isolated and pulsed with 5x10^5^ pfu/ml ultraviolet (UV)-inactivated HSV-2 for 16 hours. These TC were co-cultured for 3.5 days with CD4^+^ T cells (TC + HSV-2 CD4) isolated from the draining lymph nodes and vaginal tracts of OVX mice immunized and challenged intravaginally with HSV-2, to determine the influence of E2 on HSV-2 specific T cell responses. To determine if E2 treatment would influence non-specific CD4^+^ T cell responses, co-cultures were done with naïve CD4^+^ T cells (TC + control CD4) isolated from the spleen of uninfected OVX mice, as detailed in the Materials and Methods section. T cell responses were determined by measuring IL-17 levels (mean ± SD) in co-culture supernatants by ELISA. IL-17 levels were significantly increased in co-cultures containing TC from E2-treated mice and CD4^+^ T cells from HSV-2 challenged mice, following *in vitro* HSV-2 challenge, indicating an HSV-2 specific T cell response (Fig 3A). IL-17 levels were high in all co-cultures containing TC from E2-treated mice, regardless of whether T cells were from HSV-2 challenged mice or naïve T cells. In comparison, co-cultures containing TC from mock (OVX) mice had little to no IL-17, regardless of the source of T cells. Only in co-cultures containing CD4^+^ T cells from HSV-2 challenged mice, low levels of IL-17 were detected following *in vitro* challenge, but this was significantly lower than that seen in E2 TC co-cultures (Fig 3A). Overall, these results indicate that APCs in the E2-treated vaginal tissue are uniquely conditioned to induce differentiation of CD4^+^ T cells towards T_h_17.

Given the practical challenge of obtaining sufficient numbers of CD4^+^ T cells from HSV-2 immunized/challenged mice and the low HSV-2 specific T cell responses seen in previous experiments, we next determined if this phenomenon of E2-conditioned T_h_17 responses could be observed using a previously well-described chicken ovalbumin (OVA) peptide model with OVA-specific OT-II transgenic (Tg) CD4^+^ T cells [37]. OVX mice were implanted with E2, P4, or placebo (mock) pellets, and two-weeks later, vaginal tissue cells containing all local APCs were isolated, pulsed with OVA peptide, and co-cultured with CFSE-stained OT-II Tg CD4^+^ T cells (TC+CD4), as detailed in the Materials and Methods section. To examine T_h_1 and T_h_17 differentiation in these co-cultures, IFN-γ and IL-17 levels (mean ± SD) in culture supernatants were measured by ELISA. While there were no differences in CD4^+^ T cell proliferation between E2, P4 or mock cultures (Fig 3B), the supernatants from co-cultures containing TC from E2-treated mice had > 7 to 18-fold higher IL-17 and 12-fold higher IFN-γ levels, compared to co-cultures containing TC from P4- and mock (placebo)-treated mice (Fig 3C) (IL-17 in TC+CD4: (E2: 6297 ± 974 pg/mL; P4: 805.6 ± 82 pg/mL; mock: 349 ± 76 pg/mL); IFN-γ in TC+CD4: (E2: 996 ± 331 pg/mL; P4: 79 ± 19 pg/mL; mock: 61 ± 26 pg/mL)). Intracellular staining on day 2 of co-cultures showed that over 70% of proliferating CFSE-stained OT-II Tg CD4^+^ T cells in E2-treated vaginal TC co-cultures expressed IL-17 and ROR-γt, the master-regulator transcription factor for T_h_17 cells, showing that T_h_17 cells were the primary source of IL-17 in these co-cultures (Fig 3D). These results indicated that co-cultures utilizing OT-II Tg CD4^+^ T cells and OVA peptide could be used to examine the effect of E2 on vaginal APCs in further experiments.

The E2-dependent induction of T_h_17 responses by vaginal tissue cells has not been described previously. Given this unique observation, we examined hormone-dependent T_h_17 differentiation further. Both E2 and P4 are continually present in the reproductive tract, albeit in different ratios, throughout the different phases of the reproductive cycle [38]. We wanted to examine if the differential conditioning of vaginal APCs to induce T_h_17 responses could be observed in the vagina of mice during the normal reproductive cycle. Co-cultures were conducted with vaginal TC isolated from mice in estrus (E2-dominant) or diestrus (P4-dominant) stages, while vaginal TC from OVX mice served as a control. Vaginal TC from mice in estrus induced over 3- to 4-fold higher IL-17 levels in co-cultures compared to TC from mice in diestrus or OVX controls (Fig 3E) (Estrus: 23827 ± 2452 pg/mL; Diestrus: 8248 ± 3244 pg/mL; OVX: 5744 ± 1573 pg/mL). This suggests that although E2 is present throughout the reproductive cycle, changes in E2 levels during the estrus cycle may be sufficient to condition vaginal APCs to prime differential T_h_17 responses.

To confirm the role of E2, IL-17 levels were compared between co-cultures conducted with vaginal TC from estrogen receptor knockout (ERKO) mice, and a pooled group of WT mice at different stages of the reproductive cycle. Vaginal cells from ERKO mice induced 12-fold lower IL-17 levels compared to WT controls (WT: 6837 ± 1938 pg/mL; ERKO: 559 ± 58 pg/mL), confirming that E2 is critical for the priming of T_h_17 responses by vaginal TC (Fig 3F). Interestingly, ERKO spleen TC co-cultures contained significantly higher IL-17 levels compared to WT controls (WT: 364 ± 61 pg/mL; ERKO: 828 ± 121 pg/mL) (Fig 3F). This suggests that the E2 conditioning of APCs to induce T_h_17 responses may be limited to the vagina.

Overall, these results indicate that E2-conditioned vaginal cells induce the differentiation of CD4^+^ T cells into IL-17-producing T_h_17 cells. Furthermore, endogenous levels of E2 throughout the reproductive cycle appeared sufficient to prime vaginal APCs for induction of T_h_17 responses. Therefore, in the following experiments, we pooled mice from all stages of their normal reproductive cycle so that we could conduct experiments with larger n numbers.

### Vaginal CD11c^+^ DCs are potent inducers of T_h_17 responses

**Fig 4:**
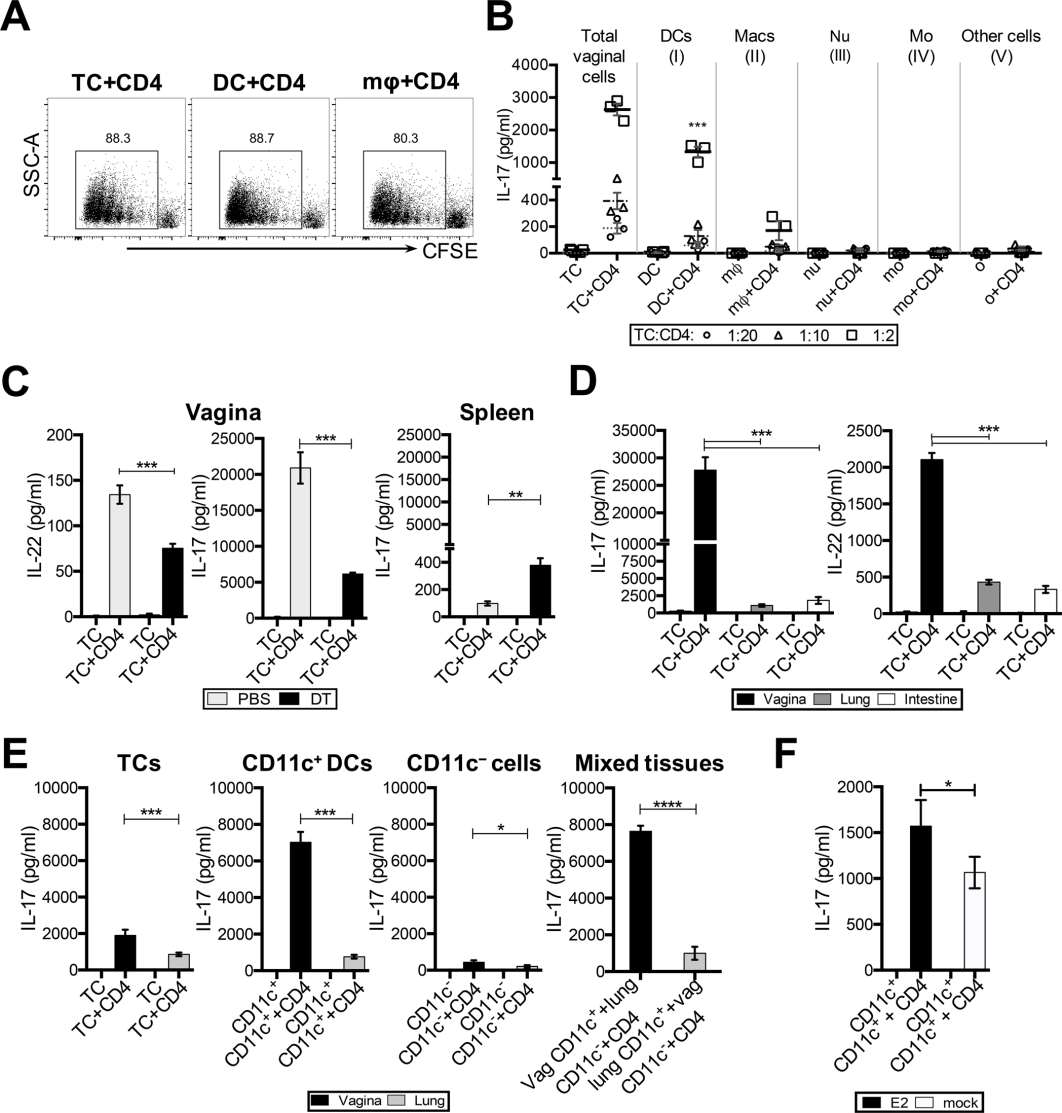
Vaginal CD11c^+^ DCs are the primary inducers of T_h_17 responses, and are more potent inducers than other mucosal DCs. Vaginal cells from WT mice (n = 13 mice) were pooled and sorted by FACS, and total vaginal cells, as well as sorted populations, were pulsed with OVA peptide and co-cultured with 5x10^5^ cells/ml OT-II Tg CD4^+^ T cells at the indicated ratios. (A) CD4^+^ T cell proliferation in total vaginal tissue cell co-cultures, CD11c^+^ DC co-cultures and macrophage co-cultures. (B) IL-17 levels in co-culture supernatants were measured by ELISA and represented as mean±SD of three separate wells per co-culture condition. Statistical analysis was done by one-way ANOVA, to calculate significant differences in IL-17 levels between total vaginal co-cultures and the indicated cell-specific co-cultures at each given ratio of APCs:T cells. Data is representative of two separate experiments with similar results. (C) CD11c DTR mice (n = 5 mice/group) were treated with 400ng DT (200ng IVAG + 200ng IP) or PBS, and 18h later, vaginal tissues and spleen from each group were pooled, and 5x10^5^ tissue cells/ml were pulsed with OVA peptide, and co-cultured with OT-II Tg CD4^+^ T cells in a 1:1 ratio for 3.5 days. IL-17 and IL-22 levels in vaginal co-cultures, and IL-17 levels in spleen tissue co-cultures, were compared between DT-treated and PBS-control groups. Data is represented as mean±SD of three separate culture wells from one of two separate experiments with similar trends, and significance was calculated by two-way ANOVA (** p<0.01, *** p<0.001). (D) The vagina, lung and small intestine from WT mice were isolated, pooled (n = 7 mice) and processed into a cell suspension. Total tissue cells (5x10^5^ cells/ml) were cultured alone (TC) or co-cultured with 5x10^5^ OT-II Tg CD4^+^ T cells for 3.5 days, and IL-17 and IL-22 levels in culture supernatants were measured by ELISA. Data is represented as mean±SD of three separate culture wells from one of three separate experiments with similar trends, and significance was calculated by one-way ANOVA (*** p<0.001). (E) CD11c^+^ DCs and CD11c^−^ cells were sorted by FACS from the lungs and vagina of WT mice, and total tissue cells (5x10^5^ cells/ml), or purified cells (2.5x10^5^ cells/ml), were OVA peptide pulsed and co-cultured with OT-II Tg CD4^+^ T cells (5x10^5^ cells/ml). For heterologous mixed co-cultures, CD11c^+^ cells (2.5x10^5^ cells/ml) from the vagina or lung were mixed with CD11c^−^ cells (2.5x10^5^ cells/ml) from the other tissue, pulsed with OVA peptide, and co-cultured with CD4^+^ T cells (5x10^5^ cells/ml). IL-17 levels in supernatants were measured by ELISA. Significance was calculated by comparing mean±SD of three separate co-culture wells per condition, by one-way ANOVA (* p<0.05, ** p<0.01, *** p<0.001, **** p<0.0001). Data is representative from two separate experiments with similar trends. (F) CD11c+ DCs (2.5x10^5^ cells/ml) sorted from the vaginal tissues of OVX mice implanted with E2 or placebo (mock) pellets (n = 20 mice/group), were pulsed with OVA peptide, and co-cultured with CD4+ T cells (5x10^5^ cells/ml) for 3.5 days. IL-17 levels in co-culture supernatants were measured by ELISA. Significance was calculated by comparing mean±SD of 6 replicates per condition, by a two-tailed unpaired T test with Welch’s correlation (* p = 0.026).

Next, we wanted to identify the specific APC populations in the vagina that were responsible for priming these T_h_17 responses. DCs, broadly classified as CD11c^+^ cells, macrophages (CD11c^−^ CD11b^+^ F4/80^+^ Gr-1^−^), neutrophils (CD11c^−^ CD11b^+^ F4/80^−^ Gr-1^+^), monocytes (CD11c^−^ CD11b^+^ F4/80^+^ Gr-1^+^) and other cells (CD11c^−^ CD11b^−^), were sorted by FACS, pulsed with OVA peptide, and co-cultured in different ratios with 1x10^5^ OT-II Tg CD4^+^ T cells. Vaginal TC, CD11c^+^ DCs, and macrophages induced a similar degree of CD4^+^ T cell proliferation in co-cultures (Fig 4A). However, cytokine analysis showed that vaginal CD11c^+^ DCs were the primary inducers of IL-17 from T cells in co-cultures (1331 ± 276 pg/mL at 1:2 ratio, Fig 4B). Macrophages also induced IL-17 levels, albeit 8-fold less in magnitude (170 ± 125 pg/mL at 1:2 ratio) compared to DCs. Neutrophils, monocytes, and other cells did not induce any detectable IL-17, showing that DCs and macrophages may be the sole inducers of T_h_17 differentiation *in vitro*.

To confirm that CD11c^+^ DCs were the primary inducers of T_h_17 responses in the genital tract, CD11c^+^ cells were depleted in the vagina by injecting CD11c-DTR mice with 400ng (200ng IP + 200ng intravaginally) of diphtheria toxin (DT); a separate group of CD11c-DTR mice treated with PBS were used as controls. Vaginal TC from both these groups were pulsed with OVA peptide, and co-cultured with OT-II Tg CD4^+^ T cells. Since T_h_17 cells are known to produce IL-22 in addition to IL-17 [39], we measured both in co-culture supernatants. Co-cultures with TC from CD11c-depleted mice (DT group) contained approximately 3.5-fold lower levels of IL-17 (PBS: 20895 ± 3766 pg/mL; DT: 6152 ± 341 pg/mL), and 2-fold lower levels of IL-22 (PBS: 134 ± 17 pg/mL; DT: 75 ± 8 pg/mL), compared to TC from CD11c-intact mice (PBS group) (Fig 4C). Similar to the observations in Fig 3E, co-cultures with splenocytes from CD11c-depleted mice showed an increase in IL-17 compared to controls.

These findings confirm the role of vaginal CD11c^+^ DCs in priming T_h_17 responses (Fig 4B), and indicate tissue-specific differences in the propensity of APC populations to prime T_h_17 CD4^+^ T cell responses.

Given the distinct differences in T_h_17 responses between vaginal and spleen co-cultures, we examined whether the potential of vaginal TC or CD11c^+^ DCs to prime T_h_17 responses is comparable to total cells or CD11c^+^ DCs from other mucosal tissues, such as the lung or small intestine. Vaginal, lung and small intestine TC (containing all their respective APC populations), were pulsed with OVA peptide, co-cultured with OT-II Tg CD4^+^ T cells, and T_h_17 cytokines were examined in co-culture supernatants. There were no differences in CD4^+^ T cell proliferation among co-cultures of TC+CD4 from all three mucosal tissues; however, vaginal co-cultures contained over 15-fold higher levels of IL-17 (Vagina: 27780 ± 4051 pg/mL; Lung: 1086 ± 326 pg/mL; Intestine: 1827 ± 878 pg/mL) and 4-fold higher levels of IL-22 (Vagina: 2105 ± 157 pg/mL; Lung: 430 ± 54 pg/mL; Intestine: 331 ± 81 pg/mL), compared to lung or intestine TC co-cultures (Fig 4D), suggesting mucosal tissue-specific differences in the ability of the respective APC populations to prime CD4^+^ T cell responses.

To directly compare the abilities of vaginal and lung CD11c^+^ DCs to prime T_h_17 responses, CD11c^+^ and CD11c^−^ cells from both these tissues were sorted, peptide pulsed, and co-cultured with OT-II Tg CD4^+^ T cells at a 1:2 ratio of APC:CD4. Vaginal CD11c^+^ DCs induced 10-fold higher IL-17 levels compared to lung CD11c^+^ DCs (Vagina: 7017 ± 577 pg/mL; Lung: 764 ± 105 pg/mL) (Fig 4E). Vaginal total cells (Vagina: 1901 ± 315 pg/mL; Lung: 858 ± 102 pg/mL) and CD11c^−^ cells (Vagina: 436 ± 109 pg/mL; Lung: 206 ± 70 pg/mL) also induced significantly higher levels of IL-17, although not as striking as CD11c^+^ DCs. In order to examine the role of CD11c^−^ cells in conditioning the vaginal and lung CD11c^+^ cells, CD11c^+^ cells from each mucosa were mixed with CD11c^−^ cells from the heterologous mucosa in APC:T cell co-cultures. We found that CD11c^−^ cells did not influence the ability of vaginal or lung CD11c^+^ cells to prime T_h_17 responses (Fig 4E: Mixed tissues). Vaginal and lung CD11c^+^ DCs retained their respective ability to induce T_h_17 responses, regardless of the tissue source of CD11c^−^ cells (Vagina CD11c^+^ + Lung CD11c^−^: 7633 ± 307 pg/mL; Lung CD11c^+^ + Vagina CD11c^−^: 994 ± 351 pg/mL). This suggests that mucosal DCs are programmed within their respective tissue microenvironment, and a short-term co-culture with cells from other tissues is not sufficient to change their propensity.

To determine if increased T_h_17 responses seen in E2-treated mice were primarily due to the effect of E2 on CD11c^+^ DCs, CD11c^+^ DCs from the vaginal tracts of E2- and placebo (mock)-treated mice were sorted, peptide pulsed, and co-cultured with OT-II Tg CD4^+^ T cells. Vaginal CD11c^+^ DCs from E2-treated mice induced significantly higher IL-17 levels compared to mock controls (E2: 1571 ± 284 pg/mL and mock: 1064 ± 171 pg/mL) (Fig 4F).

Overall, these results indicate that E2 conditions CD11c^+^ cells in the vagina to become the primary inducers of T_h_17 responses, and this phenomenon is unique to vaginal mucosa.

### E2 influences vaginal DCs to induce T_h_17 responses through an IL-1-dependent, but IL-6-independent mechanism

**Fig 5:**
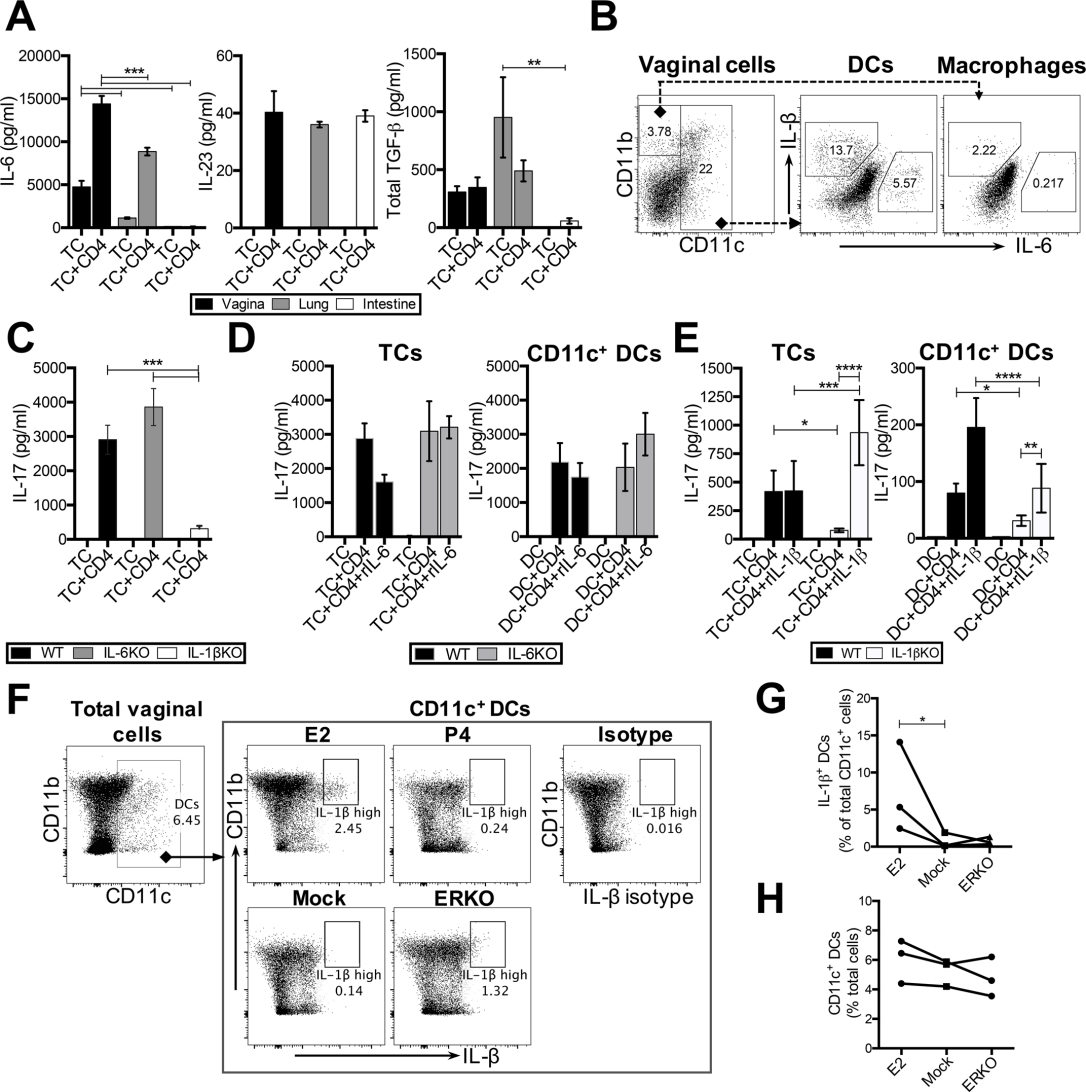
E2 conditions vaginal DCs to induce T_h_17 responses through an IL-1-dependent pathway. (A) Tissue cells from the vagina, lung and intestine were pulsed with OVA peptide and co-cultured with CD4^+^ T cells (TC+CD4). IL-6, IL-23 and TGF-β were measured in co-culture supernatants by ELISA. (B) Intracellular staining of vagina co-cultures on day 2 of co-culture to examine IL-1β and IL-6 production by vaginal DCs (CD11c^+^ cells) and macrophages (CD11c^−^ CD11b^+^ F4/80^+^ Gr-1^−^). (C) IL-17 levels compared among vaginal TC co-cultures from IL-6 KO, IL-1β KO and WT control mice. (D) IL-17 levels compared in co-cultures conducted with TC or CD11c^+^ DCs purified from the vagina of IL-6 KO mice and WT controls. 40 ng/ml of rIL-6 was added to co-cultures as indicated on X-axis. (E) IL-17 levels were compared in co-cultures conducted with TC or CD11c^+^ DCs purified from the vagina of IL-1β KO mice and WT controls. 100 ng/ml rIL-1β was added to co-cultures as indicated on X-axis. (F) Vaginal cells were cultured overnight without any stimulation, and intracellular staining was conducted to identify IL-1β production in CD11c^+^ DCs from OVX mice implanted with E2, P4 or placebo (mock) pellets. G) Matched experimental data showing the proportion of IL-1β^+^ DCs. Significance was calculated by a ratio paired t test and * p = 0.0284. (H) CD11c^+^ DCs compared among E2-, P4- or placebo (mock)-treated mice from three independent experiments. Data for all cytokine measurements is represented as mean±SD of three separate co-culture wells. Data is a representative of at least two separate experiments with similar results, and significance was calculated by two-way ANOVA. (* p<0.05, ** p<0.01, *** p<0.001, **** p<0.0001).

Next, we wanted to examine the factors responsible for priming T_h_17 responses in vaginal tissue co-cultures. A cytokine microenvironment containing IL-6, TGF-β and IL-23 is considered essential for priming canonical T_h_17 responses [40]. However, alternative pathways involving IL-1 signalling in combination with IL-6, IL-21 and IL-23 have also been described [41]. To determine the key factors for T_h_17 responses induced by vaginal CD11c^+^ DCs, IL-6, IL-23, TGF-β and IL-1β levels were measured in supernatants of TC+CD4 co-cultures from vagina, lung and intestines (Fig 5A). There were no significant differences in IL-23, and TGF-β levels were highest in lung co-cultures (Fig 5A). However, vaginal TC alone constitutively produced high levels of IL-6 (4755 ± 1223 pg/mL) (Fig 5A), and this was further enhanced in vaginal TC+CD4 co-cultures (14407 ± 1602 pg/mL). Both lung (8857 ± 766 pg/mL) and intestinal (136 ± 9 pg/mL) co-cultures produced significantly lower levels of IL-6. While we were unable to detect IL-1β secreted into co-culture supernatants by ELISA, intracellular staining showed that IL-1β and IL-6 were both produced by vaginal CD11c^+^ DCs and to a much less extent by macrophages (Fig 5B), suggesting that either one or both cytokines may play an important role in induction of T_h_17 responses.

To determine the role of IL-6 and IL-1β in the induction of T_h_17 responses, vaginal TC (Fig 5C) or CD11c^+^ cells sorted from reproductive-cycle matched WT controls, IL-6 KO mice (Fig 5D) and IL-1β KO mice (Fig 5E) were co-cultured with OT-II Tg CD4^+^ T cells. Vaginal TC and CD11c^+^ DCs from IL-6 KO mice (Fig 5C and 5D) were fully capable of priming T_h_17 responses in co-cultures, and the addition of exogenous rIL-6 did not significantly affect IL-17 levels in co-cultures (Fig 5D). However, T_h_17 responses were significantly impaired in co-cultures containing vaginal TC or CD11c^+^ DCs from IL-1β KO mice (Fig 5C and 5E), and this effect was reversed by the addition of exogenous rIL-1β (Fig 5E). These results show that IL-1, but not IL-6 signalling, was essential in vaginal DCs for the induction of T_h_17 responses.

Next, we wanted to determine the link between IL-1 and E2 in vaginal DC conditioning. Vaginal TC were isolated from OVX mice treated with E2, P4, or placebo (mock) pellets, and ICS was used to examine whether E2 induced IL-1β production within vaginal DCs. E2 treatment induced a unique, IL-1β^high^ CD11c^+^ DC population that was decreased in both P4-treated, ERKO and placebo-treated (mock) controls (Fig 5F). Data compiled from three separate experiments showed that this decrease in the IL-1β^high^ CD11c^+^ DC population was consistent and significant (Fig 5G). Furthermore, we did not observe any significant differences in the proportion of total CD11c^+^ DCs among E2, mock and ERKO mice (Fig 5H), suggesting that the differences in T_h_17 responses among hormone-treated mice is primarily due to increased frequency of IL-1β^high^ CD11c^+^ DCs in the E2-treated vaginal tract and not due to altered proportion of CD11c^+^ DCs.

Overall, these results show that E2 can directly condition vaginal DCs to become potent inducers of T_h_17 responses, through an IL-1-dependent pathway.

### E2-mediated conditioning of DCs is not altered by TLR ligands

**Fig 6:**
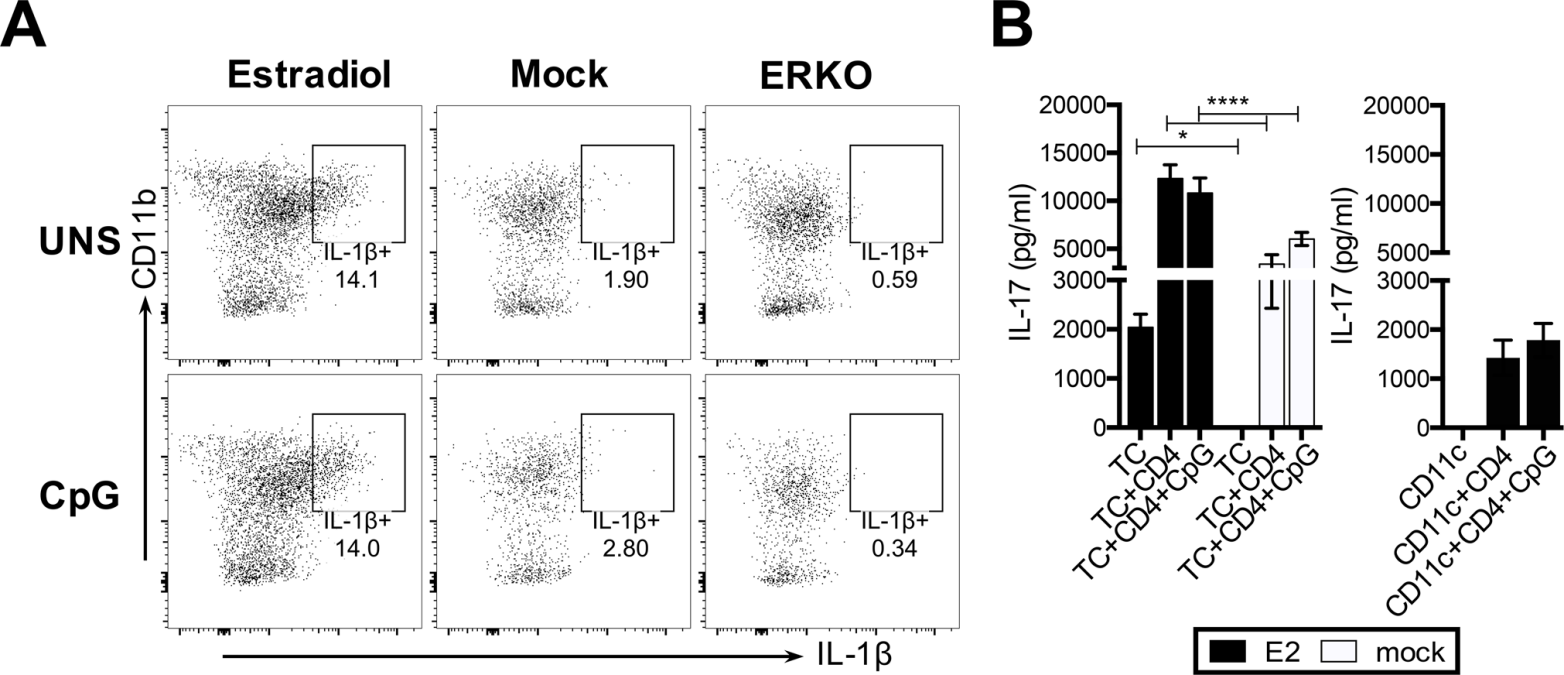
E2 conditioned T_h_17 responses are not altered by the addition of CpG. (A) OVX mice were implanted with E2 or placebo (mock) pellets, and 14 days later, vaginal cells were isolated and incubated overnight unstimulated (UNS), or stimulated with the TLR ligand CpG. ICS was conducted to examine the expression of IL-1β in CD11c+ vaginal DCs. (B) Vaginal cells from E2- or placebo (mock)-treated mice, and CD11c+ DCs flow sorted from E2-treated mice were pulsed with OVA peptide in the presence or absence of CpG for 12h, and co-cultured with OT-II Tg CD4+ T cells for 3.5 days. IL-17 levels in supernatants were measured by ELISA. Significance was calculated by comparing mean±SD of three separate co-culture wells per condition, by two-way ANOVA (* p<0.05, **** p<0.0001), and data is representative of two similar experiments.

While the influence of E2 on DC conditioning was clearly observed in previous experiments, whether the activation of DCs by viral PAMPs also played a role in the ability of DCs to induce T_h_17 responses was less clear. Previously, others have shown that TLR9 expressed in cDCs and pDCs recognizes HSV-2 dsDNA [42, 43]. Hence, we conducted an experiment to test whether a known TLR9 ligand, CpG oligodeoxynucleotides (CpG), could affect IL-1β production and T_h_17 responses primed by vaginal DCs. TC from ERKO or OVX mice treated with E2 or placebo (mock) pellets were stimulated with CpG as described in the Materials and Methods section, and ICS was used to examine IL-1β expression. As expected, IL-1β expression was high in CD11c^+^ CD11b^+^ DCs from E2-treated mice, compared to mock controls and ERKO mice. Treatment with CpG did not have any effect on IL-1β expression in any of the groups (Fig 6A). To further examine if treatment with CpG could influence T_h_17 responses primed in TC co-cultures, vaginal TC from E2- or placebo (mock)-treated mice were pulsed with OVA peptide in the presence or absence of CpG and co-cultured with OT-II Tg CD4^+^ T cells, and IL-17 levels in supernatants were measured by ELISA. Consistent with the observations seen in IL-1β expression (Fig 6A), CpG did not significantly influence T_h_17 responses primed by TC from E2- or placebo (mock)-treated mice (Fig 6B). Co-cultures conducted with pure CD11c^+^ DCs sorted from E2-treated mice and OT-II Tg CD4+ T cells, confirmed that IL-17 production by CD4^+^ T cells was not affected by CpG. Overall, these results indicated that viral pathogen-associated molecular patterns (PAMPs), such as CpG, do not significantly influence E2-conditioned T_h_17 responses primed by vaginal DCs.

### IRF4 is not essential for vaginal T_h_17 responses

**Fig 7:**
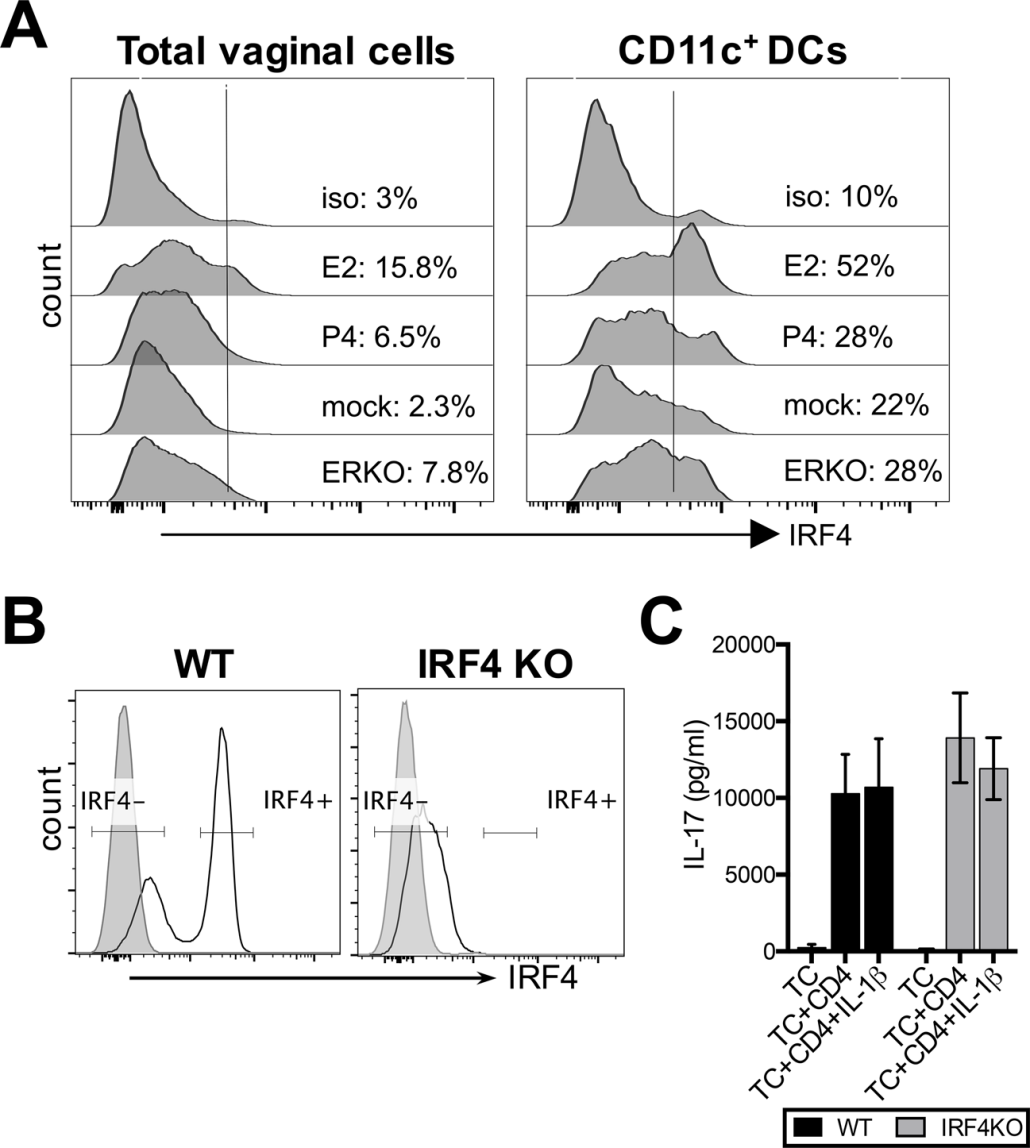
IRF4 expression is not critical for E2-mediated priming of vaginal T_h_17 responses. **(**A) Vaginal cells from WT OVX mice implanted with E2, P4 or placebo (mock) pellets for 14 days were cultured in media overnight without any stimulation (12h), and stained for antibodies against IRF4 and DCs (CD11c, CD11b). ICS was conducted according to protocols in Materials and Methods to identify IRF4 expression in total vaginal cells and vaginal CD11c^+^ DCs. (B) Spleens from IRF4 KO mice and their WT littermates were isolated, and cultured overnight without stimulation. IRF4 expression in CD4^+^ T cells was compared between IRF4 KO mice and WT littermates by ICS. (C) Vaginal cells from reproductive cycle stage matched WT and IRF4 KO mice were pulsed with OVA peptide, and co-cultured with OT-II Tg CD4+ T cells for 3.5 days. IL-17 levels in co-cultures were measured by ELISA, and expressed as mean±SD of three replicate wells from one of two different experiments. Analysis was conducted by two-way ANOVA.

Previously, others have shown that human and murine mucosal DCs expressing IRF4, may play a central role in mucosal T_h_17 differentiation [44, 45]; furthermore, *in vitro*, E2 could induce IRF4 expression in bone-marrow-derived DCs (BMDCs) [46]. Therefore, we wanted to examine whether IRF4 was critical for induction of T_h_17 responses primed by vaginal tissue cells. To examine whether E2 directly induces IRF4 expression in vaginal DCs *in vivo*, IRF4 expression in the vagina of OVX mice treated with E2, P4 or placebo (mock) pellets was examined. E2 treatment led to approximately 2-fold higher IRF4 expression in freshly isolated total vaginal cells (E2: 15.8%; P4: 6.5%; mock: 2.3%), and CD11c^+^ DCs (E2: 52%; P4: 28%; mock: 22%), compared to P4- or placebo (mock)-treatments (Fig 7A). ERKO mice showed a similar frequency of IRF4-expressing total cells and DCs compared to placebo (mock)-treated controls (Fig 7A). This indicates that in agreement with previous *in vitro* studies [46], E2 can directly induce IRF4 expression in vaginal DCs *in vivo*.

Next, we examined whether IRF4 plays a critical role in T_h_17 responses primed by vaginal DCs, by conducting APC-T cell co-cultures with vaginal TC from IRF4 KO mice and comparing them with WT control mice. Somewhat surprisingly, there were no significant differences in IL-17 levels between co-cultures with vaginal cells from IRF4 KO or WT controls (Fig 7C). The IRF4 KO phenotype was confirmed by ICS to rule out technical issues with IRF4 KO mice (Fig 7B).

Overall, this shows that while E2 can directly upregulate IRF4 in vaginal DCs *in vivo*, IRF4 does not appear to play a critical role in DC priming of T_h_17 responses.

### IL-17 KO mice are more susceptible to intravaginal HSV-2 re-exposure due to lower IFN-γ responses

**Fig 8:**
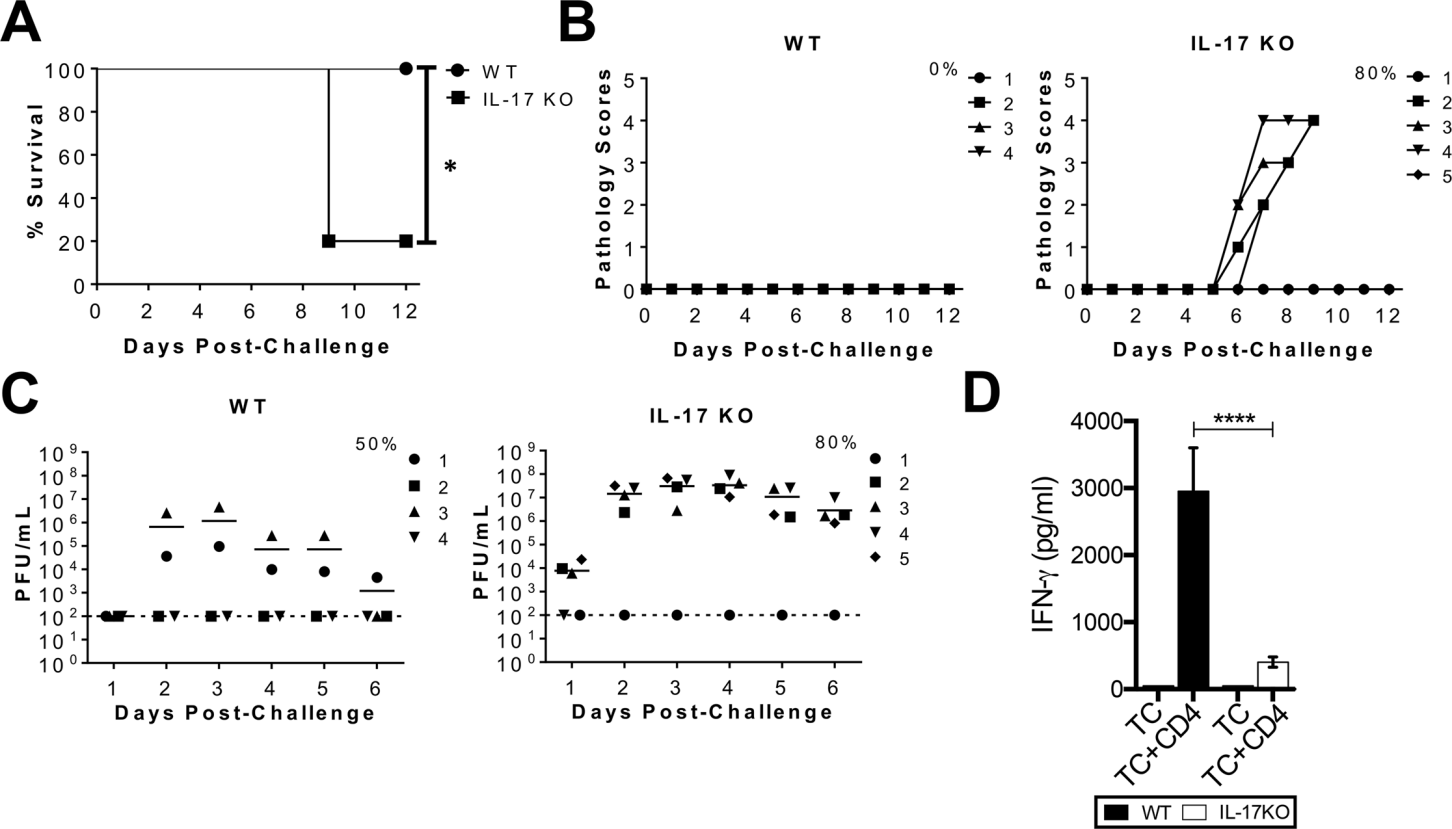
IL-17 KO mice are more susceptible to intravaginal HSV-2 challenge. OVX IL-17 KO (n = 5 mice) and WT mice (n = 4 mice) were intravaginally exposed to a sub-lethal dose of HSV-2 333 (10^2^ pfu/mouse), and 8 weeks later, intravaginally challenged with a lethal dose of HSV-2 333 (5x10^3^ pfu/mouse). (A) Survival curves for IL-17 KO and WT mice showing percentage of mice that survived challenge. Significance in difference in survival was calculated using the log-rank (Mantel-Cox) test (* p<0.05). (B) Genital pathology graded on a 1–5 scale for both groups of mice for 12 days post-challenge. Data points superimposed on X-axis indicate mice without genital pathology, and the % indicates maximum number of mice that showed pathology. (C) HSV-2 viral shedding (pfu/mL) in vaginal washes collected for 6 days post-challenge, was measured by conducting viral titers using vero cells. The dashed line indicates the lower detection limit of this assay. The % indicates maximum number of mice that shed virus between days 1 to 6 post challenge. Each symbol represents a single animal, and data points on the lower limit indicate mice that do not show detectable viral shedding in vaginal washes. The survival curves, pathology and viral titers are from a single representative of three separate experiments with similar results. (D) Vaginal tissues from stage-matched WT and IL-17 KO mice were isolated, pulsed with OVA peptide and co-cultured for 3.5 days with OT-II Tg CD4^+^ T cells. IFN-γ levels produced by vaginal tissue cells alone (TC) and co-cultures (TC+CD4) were measured by ELISA. Data is represented as mean±SD of three co-culture wells, and is a representative of three separate experiments with similar trends. Significance was calculated by two-way ANOVA (**** p<0.0001).

Since our results demonstrated that better protection in E2-treated mice post-challenge (Figs 1 and 2) coincided with enhanced T_h_17 responses and E2 treatment conditioned DCs to prime T_h_17 responses (Fig 3), we wanted to examine whether IL-17 played a role in anti-viral immunity against HSV-2. Based on vaccine models against lung *Mycobacterium tuberculosis* [47], we predicted that HSV-2 exposed IL-17 KO mice would display a compromised recall memory T cell response, and would be unable to protect against subsequent intravaginal exposure to HSV-2. OVX IL-17 KO and WT mice were infected intravaginally with a sub-lethal dose (10^2^ pfu/mouse) of WT HSV-2 333. Following this primary exposure, there were no differences in the survival, pathology or viral shedding, indicating no difference in susceptibility or anti-viral responses between IL-17 KO and WT mice to primary infection. We then sought to examine whether these HSV-2 pre-exposed IL-17 KO mice would show compromised anti-viral responses following re-exposure, since this would test the efficacy of recall memory T_h_1 effector cells. Pre-exposed OVX IL-17 KO and WT mice were re-exposed, intravaginally, to a lethal dose (5x10^3^ pfu/mouse) of WT virus. 100% of WT control mice survived the lethal challenge, while only 20% of mice survived in the IL-17 KO group (Fig 8A). IL-17 KO mice also showed greater cumulative pathology (12), compared to WT controls (0) (Table 3, Fig 8B). Furthermore, 80% of IL-17 KO mice shed virus compared to 50% of WT controls (Fig 8C). These results support our hypothesis and show that IL-17 KO mice are more susceptible to HSV-2 re-exposure due to decreased efficiency of recall HSV-2 anti-viral responses compared to WT mice. Similar results were obtained with mice pre-exposed to two other sub-lethal doses of HSV-2 followed by challenge.

**Table 3:** Cumulative pathology scores for HSV-2 pre-exposed WT and IL-17 KO mice challenged with WT HSV-2.

Treatment Group (total # of mice)	PathologyScore	# of mice	# of days	CumulativePathology	Avg. Pathology per Mouse
**WT (n = 4)**	0	4	12	0	0
**IL-17 KO (n = 5)**	0	1	12	0	12
	5	4	3	60	

Cumulative pathology is calculated by denoting the number of mice with their maximum pathology score and the number of days that score was observed for each group. This takes into consideration that each mouse in a group can reach varying degrees of pathology through the experiment. Average pathology score per mouse was calculated by dividing the sum of cumulative pathology by total number of mice. Data representative of three separate experiments with similar results.

Next, we wanted to examine whether the decreased efficiency of memory recall responses in IL-17 KO mice corresponded to an intrinsic impairment in priming T_h_1 responses by vaginal APCs. Vaginal TC from estrus cycle stage-matched IL-17 KO mice and WT controls were pulsed with OVA peptide, and co-cultured with OT-II Tg CD4^+^ T cells; IFN-γ levels were measured in co-culture supernatants after 3.5 days. Vaginal TC from IL-17 KO mice were significantly impaired at inducing IFN-γ^+^ in co-cultures compared to WT TC (WT: 2951 ± 650 pg/mL; IL-17 KO: 404 ± 77 pg/mL) (Fig 8D). These results indicate that IL-17 may indeed enhance anti-viral T_h_1 responses in the genital tract.

## Discussion

In the current study, we provide evidence for a novel mechanism whereby E2 enhances anti-viral responses in the genital tract by modulating the functions of vaginal DCs. We demonstrated that better protection in E2-treated mice coincided with accelerated and greater T_h_1 and T_h_17 responses in the vagina post-intravaginal HSV-2 challenge. E2 treatment directly conditioned vaginal DCs to become potent inducers of T_h_17 responses, and this ability of E2 to direct T_h_17 responses was dependent on the induction of IL-1β in vaginal CD11c^+^ DCs. Furthermore, this novel mechanism of E2-mediated conditioning was specific to vaginal DCs, as it was not observed in DCs isolated from spleen or other mucosal tissues including the intestine and lung. To the best of our knowledge, this is the first study demonstrating that E2 can directly regulate T-cell mediated adaptive anti-viral immunity in the female genital tract by modulating DC functions.

While others and we have previously reported that the presence of E2 during immunization can improve anti-viral protection in HSV-2 vaccine models [10, 12, 14, 15], the underlying mechanism has remained largely unknown. The current study was designed to address this, and shows that the unique feature of the E2-mediated enhanced protection against HSV-2 was through the induction of T_h_17 responses in the genital tract. While the contribution of IFN-γ^+^ CD4^+^ T_h_1 cells to HSV-2 anti-viral responses is well understood, this is the first report indicating that T_h_17 cells may augment the HSV-2 anti-viral T_h_1 responses. The efficiency of T-cell immunity against HSV-2 is best tested in recall responses post-challenge, and this effect was clearly seen in IL-17 KO mice pre-exposed to HSV-2, that were impaired in resolving intravaginal viral challenge compared to WT controls (Fig 8).

Our results indicated that the absence of IL-17 in KO mice resulted in overall decreased efficiency of HSV-2 memory recall responses. However, the exact mechanism of how IL-17 could affect anti-viral responses remains to be elucidated. Comparison of DCs from IL-17 KO mice with normal WT mice in our studies indicated that DC conditioning may be altered in absence of IL-17. This is in agreement with another study where, in a lung *C*. *muridarum* infection model, neutralization of IL-17 significantly impaired DC functions [48] by downregulating levels of IL-12 production and decreasing MHCII and CD40 expression on DCs. The DCs produced higher levels of IL-10 in absence of IL-17 and induced higher IL-4, skewing the immune responses toward a T_h_2 phenotype instead of typical T_h_1 response [48]. Thus, further studies are necessary to examine whether similar effects of IL-17 might exist in the *in vivo* HSV-2 model. We are currently examining the mechanism by which IL-17 can influence the conditioning of vaginal DCs *in vivo*.

The T_h_17 responses shown in our study were induced by vaginal DCs through an IL-1-dependent, but IL-6-independent pathway (Fig 5C, 5D and 5E). While IL-6, along with IL-23 and TGF-β, is required for the canonical pathway of T_h_17 differentiation [39], IL-6-independent T_h_17 responses have also been identified in mucosal tissues [41, 49]. In the lamina propria of the small intestine, the IL-1β-IL-1R pathway, but not IL-6, has been shown to be essential for the differentiation of steady state T_h_17 cells in response to the microflora [50]. Furthermore, while IL-6-dependent T_h_17 responses were critical for the clearance of *C*. *rodentium* [51]¸ IL-6-independent T_h_17 cell responses were important for the resolution of *H*. *polygyrus* infection [52]. This suggests that the nature of antigens, and/or the local cytokine milieu, can determine the pathways of CD4^+^ T cell differentiation. Our study showed that while IL-1β and IL-6 were both produced by vaginal DCs, IL-1β was required, while IL-6 was dispensable, for vaginal T_h_17 responses. Furthermore, our results showed that these T_h_17 responses were induced by direct conditioning of vaginal DCs by E2 to express high levels of IL-1β. In order to examine the pathway/s that link E2 to IL-1β production and T_h_17 differentiation, we examined intracellular factors that are induced by E2 and involved in T_h_17 differentiation. Others have shown that IRF4 expression in DCs was critical for the generation of lung and intestinal T_h_17 responses [44, 45]. Additionally, E2 was found to directly induce IRF4 expression in bone marrow DCs [46]. Hence, we sought to examine whether IRF4 is integral to the pathway of T_h_17 responses primed by vaginal DCs. While E2 did indeed upregulate IRF4 expression in vaginal DCs *in vivo* (Fig 7A), unlike the observations in lung or intestinal DCs [44, 45], IRF4 was dispensable for T_h_17 differentiation, as evident by intact IL-17 levels in vaginal TC co-cultures conducted with IRF4 KO and WT mice (Fig 7C). While, it is likely that other IRFs may compensate for IRF4 in vaginal DCs [53], E2 could also upregulate inflammatory mediators, including IL-1β in DCs required for induction of Th17 responses through other pathways [54]. Furthermore, IRF4 may be involved in other immune functions that influence DC functions. Studies have shown that while IRF4 was dispensable for the development of skin-derived DCs, it was crucial for their CCR7-mediated migration to the draining lymph nodes [55]. Therefore, further *in vivo* studies may provide more comprehensive information if there are other IRF-4 related mechanisms involved in DC mediated enhancement of HSV-2 anti-viral responses.

In this study, we examined DC populations by gating CD11c^+^ cells in the lung, vagina, and small intestine. Most studies that examine DCs in mucosal tissues use a panel of cell surface markers such as CD11c, MHCII, CD8α, CD103, F4/80 and CD205 [21, 22, 56–60] to identify subsets of DCs. We chose CD11c as the primary marker to define DC populations in all tissues in order to make equivalent comparisons across different mucosal tissues. We reasoned that although this would lead to inclusion of distinct APC subsets that are unique for each mucosa, it would allow us to compare the functions of a natural mix of APCs present in each mucosa, reflecting the normal immune responses in that tissue and providing an understanding of the overall differences that exist among different mucosal tissues. On the other hand, this strategy limits comparison of APC subsets that are functionally distinct. For example, other studies examining CD11c^+^ DCs in the lung [56] have found that under steady state conditions, CD11b^−^ MHCII^−^ alveolar macrophages also express CD11c, and these cells may suppress infiltration of migratory DC populations into the lung lamina propria (LP) [61, 62]. These alveolar macrophages likely represent the primary CD11c^+^ population in the lung isolates used in our experiments. Therefore, there are clear limitations to the conclusions from our study, with the results mainly reflecting the functions of overall mucosal APCs.

Although a number of groups have examined the functional relevance of T_h_17 responses in reproductive tract infections, its role under homeostatic conditions has not been examined. IL-17 has been shown to be an important part of the immune response to *N*. *gonhorreae* and *C*. *albicans* infections [35, 36, 63]. Vulvovaginal fungal infections affect 70–75% of women, and these infections have been correlated with the E2-dominant phase of the reproductive cycle [63–65]. Therefore, pre-programming of DCs to induce a T_h_17 response under the influence of E2 may represent an evolutionary adaptation for protecting the reproductive tract against these infections. The role of T_h_17 responses in viral infections is relatively unclear. Two previous studies have suggested that IL-17 may not have a direct protective role in vaginal HSV-2 infection [66, 67]. However, both of these studies utilized MPA, a P4 derivative, to make mice susceptible to HSV-2. Previous studies, including our own, have shown that MPA can significantly downregulate endogenous hormone levels, including E2, and decrease mucosal anti-viral responses to HSV-2 [11, 68]. This may have precluded an accurate assessment of the contribution of T_h_17 cells in HSV-2 anti-viral responses. As seen in the current study, the hormonal environment can have a profound effect on the induction of adaptive immune responses. Therefore, anti-viral immune responses in the female genital tract need to be examined under clearly defined hormonal conditions.

Our study showed that T_h_17 responses coincide with augmented anti-viral immunity in E2-treated mice, and further studies are needed to demonstrate the underlying mechanism. In a pulmonary *M*. *tuberculosis* vaccination model where, like anti-HSV-2 immunity, IFN-γ produced by CD4^+^ T cells is integral to the protective immune response, the presence of IL-17 correlated with accelerated CD4^+^ T cell responses and early resolution of bacteria post-challenge [47, 69]. The T_h_17 response post-challenge was correlated with a concurrent CXCL9, CXCL10 and CXCL11 chemokine response, which was essential for the accumulation of CD4^+^ IFN-γ^+^ T cells in the lung [47]. Our observations were very similar in that E2-induced T_h_17 responses coincided with earlier and greater proportions of CD4^+^ IFN-γ^+^ T cells in the vagina post-challenge (Fig 2 and Table 2). We also showed that the presence of IL-17 was critical for priming efficient T_h_1 responses *in vitro* (Fig 8). Thus, like the *M*. *tuberculosis* study, IL-17 is likely responsible for facilitating the rapid infiltration of memory T_h_1 cells through chemokine induction in the vaginal tract. Further studies examining the chemokines and T cell subsets in the vagina post-challenge are ongoing to examine this possibility.

In summary, our study describes for the first time a mechanism by which E2 enhances anti-viral protection following vaccination in the genital HSV-2 mouse model. E2-priming resulted in vaginal APCs becoming potent inducers of T_h_17 responses, and this coincided with earlier recruitment and a greater accumulation of IFN-γ^+^ CD4^+^ T cells post-challenge. Furthermore, we demonstrated that CD11c^+^ cells in the vagina were the primary inducers of T_h_17 responses, and E2 was the critical factor that upregulated IL-1β, required for induction of T_h_17 responses. Overall, our study provides insight into a potential mechanism by which the hormonal microenvironment during immunization can regulate the induction of mucosal anti-viral T cell immune responses in the female genital tract. Hence, hormonal status should be an important consideration in the development of mucosal vaccines against sexually transmitted pathogens, to assess whether the modulation of hormonal microenvironment can potentially optimize vaccine-mediated immune responses against STIs in the female genital tract.

## Materials and Methods

### Animals

C57BL/6 mice were obtained from Charles River Laboratories Inc (Saint-Constant, QC, Canada). Chicken ovalbumin (OVA) receptor transgenic (Tg) mice (OT-II) whose CD4^+^ T cells express TCR specific for the ovalbumin 323–339 (OVA_323-339_) epitopes [37], and IL-6 knockout mice (IL-6 KO) [70], were purchased from Jackson Laboratory (Bar Harbor, Maine, USA). IL-1β KO and IL-17 KO mice were kindly provided by Dr. Yoichiro Iwakura (University of Tokyo, Minato-ku, Tokyo, Japan) [71, 72], estradiol receptor α knockout mice (ERKO) were kindly provided by Prof. P. Chambon (University de Strasbourg, France), and CD11c-DTR mice [73] were bred internally (McMaster University, Hamilton, ON, Canada). IRF4 KO mice [74] were kindly provided by Dr. Tak Wah Mak (University Health Network, Princess Margaret Cancer Centre, Toronto, ON), and bred internally (McMaster University, Hamilton, ON, Canada).

### Surgeries and treatments

Endogenous hormones were depleted by ovariectomies (OVX) according to previously published protocols [13]. Briefly, OVX mice were anaesthetised with injectable anaesthetic (150mg Ketamine/kg + 10mg Xylazine/kg body weight) and subcutaneously implanted with either 21-day release E2 (476 ng/mouse/day), or P4 (476 μg/mouse/day), or placebo pellets, purchased from Innovative Research of America (Sarasota, Florida, USA) using previously published protocols [14]. The level of serum E2 resulting from the pellets has previously been shown to correspond to that measured during the estrus cycle [75] and the P4 levels to those seen during pregnancy [76]. DCs were depleted in CD11c-DTR mice using 400ng diphtheria toxin (DT) (Sigma Aldrich, St. Louis, MO, USA) (200ng IP + 200ng intravaginal injections) treatment 18h before tissue retrieval.

### Infections

One week after implanting hormone pellets, OVX mice were immunized intranasally with 1x10^3^ TK^−^ HSV-2 or 5μg HSV-2 gD + 30μg CpG or 1x10^4^ pfu (plaque forming units) HI HSV-2 333 + 30μg CpG. The immunization was repeated two weeks later, and mice were challenged intravaginally with 5x10^3^ pfu/mouse WT HSV-2 333, according to previously published protocols [11]. Vaginal washes were collected daily post-challenge and frozen until use at -80°C. To quantify shed virus within these washes, plaque assays were conducted on Vero cells, as described before [11]. Survival and genital pathology was monitored on a five-point scale. 0: no infection, 1: slight redness of external vagina, 2: swelling and redness of vagina, 3: severe swelling and redness of vagina and surrounding tissues, 4: genital ulceration with severe redness and hair loss, and 5: severe ulceration extending to surrounding tissues, ruffled hair, hunched back and lethargy. Animals were sacrificed before they reached stage 5. For the intravaginal infection model to obtain HSV-2 specific CD4+ T cells, OVX mice were IVAG immunized with 1x10^5^ pfu/mouse of TK^-^ HSV-2, and three weeks later, challenged IVAG with 1x10^5^ pfu/mouse WT HSV-2 333. CD4^+^ T cells were isolated from the vagina and draining iliac lymph nodes from these mice 3 days post-challenge.

### Tissue isolation and co-cultures

Mucosal tissues were enzymatically digested (lung: collagenase I 150U/mL, intestine: collagenase A 0.239mg/mL and DNase I 20U/mL, vagina: collagenase A 150U/mL; (Roche Diagnostics, Mississauga, ON, Canada)) at 37°C for 1-2h as previously described [77–79]. Spleen was mechanically disrupted and ACK buffer (Sigma Aldrich, St. Louis, MO, USA) was used to lyse blood cells. Mononuclear cells were counted and cell preparations were seeded in a 96-well plate, at 5x10^5^ cells/mL or 2.5x10^5^ DCs/mL, in RPMI 1640 media supplemented with 10% FBS, 100 IU/mL penicillin, 100 μg/mL streptomycin, 1% L-glutamine, 0.1% 2-mercaptoethanol, 1x non-essential amino acids and 1x sodium pyruvate (Gibco Life Technologies, Burlington, ON, Canada). Cells were pulsed with ovalbumin 323–339 (OVA) peptide (Biomer technology, Pleasanton, CA, USA) or 5X10^5^pfu/ml of UV-inactivated HSV-2 for 6-18h. CD4^+^ T cells were magnetically sorted using CD L3TE microbeads (Miltenyi Biotec, Auburn, CA, USA) from the spleen of OT-II mice, and stained with 50μM CFSE (Sigma Aldrich, St. Louis, MO, USA) according to published protocols [80]. Peptide-pulsed tissue cells were co-cultured with CFSE-stained splenic OT-II Tg CD4^+^ T cells at a 1:1 ratio for 3.5 days at 37°C based on previously published protocols [81]. For activation of TCs with CpG, vaginal TCs were isolated from OVX mice implanted with E2 or placebo (mock) pellets for 14 days and incubated in the presence or absence of 6 ng/ml CpG ODN for 12h prior to ICS or co-culture with OT-II Tg CD4^+^ T cells. In some experiments as indicated in figure legend, 40 ng/mL rIL-6 (R&D systems, Minneapolis, MN, USA) or 100 ng/mL rIL-1β (R&D systems, Minneapolis, MN, USA) was added on the first day of co-culture. Co-culture supernatants were frozen for cytokine analysis and cells were phenotypically characterized for CD4^+^ T cell proliferation and intracellular cytokine detection by flow cytometry.

### Flow cytometry

Mononuclear tissue cells or cell fractions from co-cultures were stained with a cocktail of antibodies: [CD11c PE-Cy7, Gr-1 AF700, F4/80 APC, CD3 AF700 (eBioscience, San Diego, CA, USA), CD11b PE-CF594, I-Ab FITC (BD Biosciences, San Jose, CA, USA) CD3 BV785, CD4 BV421 (BioLegend, San Diego, CA, USA)]. For intracellular staining, on day 2 of co-culture, cells were treated with 2μl/mL Cell Stimulation Cocktail plus protein transport inhibitors (500X) (cocktail of phorbol 12-myristate 13-acetate (PMA), ionomycin, brefeldin A and monensin) (eBioscience, San Diego, CA, USA) for 12–16 h, permeabilized and fixed with BD Pharmingen™ Transcription Factor Buffer Set (BD Biosciences, San Jose, CA, USA), and stained with intracellular antibodies against cytokines (IL-17 APC, IL-6 PE (BD Biosciences, San Jose, CA, USA); ROR-γ PE, IRF4 PE, IL-1β PE, IFN-γ FITC (eBioscience, San Diego, CA, USA)). Data was acquired on a BD LSRII flow cytometer (BD Biosciences, Canada) and analyzed with FlowJo software (Treestar, Ashland, OR, USA). Flow sorting was conducted using a BD FACSAria III (BD Biosciences, San Jose, CA, USA) flow sorter to isolate the following populations: DCs (CD11c^+^ cells); macrophages (CD11c^−^ CD11b^+^ F4/80^+^ Gr-1^−^ cells); monocytes (CD11c^−^ CD11b^+^ F4/80^+^ Gr-1^+^ cells), neutrophils (CD11c^−^ CD11b^+^ F4/80^−^ Gr-1^+^ cells) and others (CD11c^−^ CD11b^−^ cells). Purity was verified by flow analysis of purified fractions on the BD FACSAria™ III, and was consistently found to be over 95%. Cells were analyzed and initially gated on forward and side scatter parameters to select total cells excluding debris or aggregates. Singlet events were selected based on forward scatter area, height and width parameters. CD3^+^ CD4^+^ T cells were selected, and IFN-γ^+^ or IL-17^+^ cells were gated to examine T_h_1 and T_h_17 populations.

### Cytokine analysis

Co-culture supernatants were assayed using DuoSet ELISA kits to measure IL-17, IL-23, IL-22 and TGF-β (R&D systems, Minneapolis, MN, USA). In some experiments, a custom MSD multiplex kit was used to measure TNF-α, IL-12, IFN-γ, IL-6, IL-17, IL-2, IL-4 and IL-10 and plates were analyzed on a Sector Imager 2400 (Meso Scale Discovery, Rockville, MD, USA).

### Statistics

Data was analyzed using GraphPad Prism 6 (GraphPad Software, San Diego, CA). The Mantel-Cox log-rank test was used to calculate significant differences in survival. One way- and two way-ANOVA were used to calculate significant differences in cytokine levels, and paired analysis for significant alterations in IL-1β expression was calculated using ratio-paired t test.

### Ethics statement

All animals in this study were housed at the McMaster Central Animal facility, and the protocols used were approved by the McMaster University Animal Research Ethics Board (AREB) as per AUP # 14-09-40 in accordance with Canadian Council of Animal Care (CCAC) guidelines.
