# Correction: Estradiol Enhances CD4^+^ T-Cell Anti-Viral Immunity by Priming Vaginal DCs to Induce T_h_17 Responses via an IL-1-Dependent Pathway

**DOI:** 10.1371/journal.ppat.1005706

**Published:** 2016-06-08

**Authors:** Varun C. Anipindi, Puja Bagri, Kristy Roth, Sara E. Dizzell, Philip V. Nguyen, Christopher R. Shaler, Derek K. Chu, Rodrigo Jiménez-Saiz, Hong Liang, Stephanie L. Swift, Aisha Nazli, Jessica K. Kafka, Jonathan Bramson, Zhou Xing, Manel Jordana, Yonghong Wan, Denis P. Snider, Martin R. Stampfli, Charu Kaushic

The tenth author's name is spelled incorrectly. The correct name is: Stephanie L. Swift.
